# Monitoring of Weekly Sleep Pattern Variations at Home with a Contactless Biomotion Sensor

**DOI:** 10.3390/s150818950

**Published:** 2015-08-03

**Authors:** Masanori Hashizaki, Hiroshi Nakajima, Kazuhiko Kume

**Affiliations:** 1 Graduate School of Pharmaceutical Sciences, Nagoya City University, Nagoya 467-8603, Japan; E-Mail: masanori_hashizaki@omron.co.jp; 2 Technology and Intellectual Property H.Q., Omron Corporation, Kyoto 619-0283, Japan; E-Mail: hiroshi_nakajima@omron.co.jp

**Keywords:** contactless, sensor, circadian rhythm, delay, weekend, schedule

## Abstract

Many people find that their sleep is restricted or disturbed by social obligations, including work. Sleep phase delays can affect an individual’s circadian rhythms on the following day and cause daytime sleepiness and/or poor performance. In this study, to examine weekly variations in sleep patterns, we analyzed sleep data for seven-day periods (from Sunday to Saturday) that had been collected from 2914 subjects (aged 20–79 years) over a total of 24,899 subject-weeks using contactless biomotion sensors. On the weekend, the subjects’ mean sleep midpoint, bedtime, and wake-up time were delayed by 40, 26 and 53 min, respectively, compared with those seen on weekdays. In addition, on weekdays, the mean difference between the maximum and median sleep midpoint ranged from 35 to 47 min among the subjects in their 20 s–70 s. The weekend delay and weekday variation in the subjects’ sleep patterns tended to decrease with age. This study detected sleep pattern disturbances on both weekdays and weekends. The serial changes in weekday bedtimes detected in this study suggest that sleep habits are influenced by changes in the temporal patterns of social activities/duties. We need further study the advantages of getting extra sleep and the disadvantages of sleep pattern disturbances in daily lifestyle.

## 1. Introduction

Sleep plays an important role in a healthy lifestyle together with nutrition and exercise. We recently developed and commercialized a contactless biomotion sensor, which easily, objectively and accurately record sleep/wake state in daily life. The data of the users are collected through an Internet system, which enables us to gather large-scale data. With this innovative sensor technology, we are analyzing sleep data at epidemiologic level. In order to keep health, we need to have proper sleep, dietary, and exercise habits. However, previous studies have found that adults tend to wake up later on weekends than on weekdays [1,2,3]. Getting less sleep on weekdays and compensating for this on the weekend are associated with the nighttime use of electronic devices such as cellphones, computers, and televisions [4]. Many individuals restrict the amount of sleep they get on weekdays because of the demands of modern lifestyles [5]. As such, people get insufficient sleep on weekdays and accumulate a sleep debt throughout the week, and therefore they frequently sleep for longer on the weekend.

A previous study based on the Munich Chronotype Questionnaire (MCTQ) found that the distributions of mid-sleep (MS) (MS = bedtime + (wake-up time − bedtime)/2) and sleep duration differ between work days and free days, and that such variations are affected by age and social habits [6]. Wittmann advocated the concept of social jetlag; *i.e.*, that discrepancies between biological and social time can have adverse effects, and reported that an index of social jetlag (ΔMS = |MSfreedays − MSworkdays|) was correlated with chronotype (MSFsc: sleep-corrected MSfreedays = MSfreedays − (sleep duration on free days − sleep duration on workdays)/2) [7]. A recent study found that the mode of ΔMS decreases with age in both men and women [8]. Sleep patterns are affected by social schedules, but the impact of particular sleep patterns depend on an individual’s chronotype [3].

Sleeping for longer on the weekend can delay an individual’s circadian rhythms and affect their subsequent sleep and daytime functions. In a previous study, the administration of melatonin counteracted the phase delay of endogenous melatonin onset, increased the severity of daytime sleepiness, and decreased sleep onset latency on Sundays [9]. In another study, subjects that delayed their sleep schedules on the weekend experienced less sleepiness and displayed a longer sleep onset latency period on Sunday nights, but exhibited lower cognitive performance and overall mood ratings on Monday mornings compared with subjects who maintained their weekday sleep schedule on weekends [10]. Similar effects have also been observed in subjects that went to bed at their normal bedtimes, but woke up later on weekends [11].

In previous studies, questionnaires were used to assess variations in weekly sleep patterns. The latter questionnaires assessed sleep patterns based on the subjects' usual sleep schedules, and thus, the data obtained were not objective. In addition, no previous study has examined the intra-weekday variation in sleep schedules. The aim of this study was to assess the variation in weekly sleep patterns by analyzing sleep data that had been obtained at subjects’ homes using contactless biomotion sensors and then uploaded to a web-based application.

## 2. Subjects and Methods

### 2.1. Subjects

**Figure 1:**
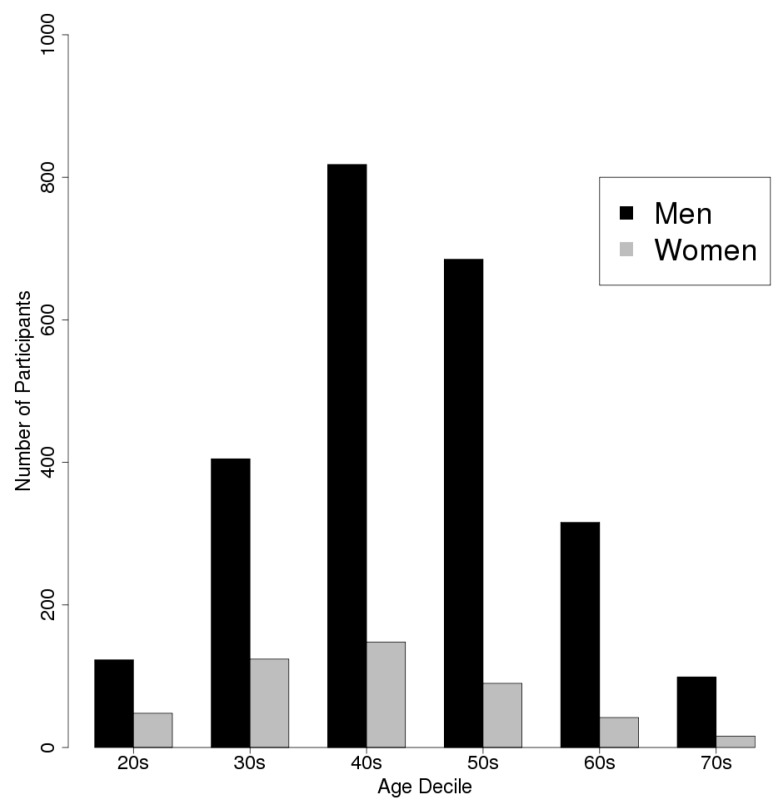
Histogram showing the age distribution of the participants according to gender (Black bars, men; gray bars, women).

The subjects were selected from a database of 6090 individuals who had bought contactless biomotion sensors (HSL-101, Omron Healthcare Co., Ltd, Kyoto, Japan) and registered with a web-based health data application. The measurement period ran from May 2012 to June 2014. We excluded individuals who were aged less than 20- or over 80-years-old because of the number of participants. Data were extracted for 7-day periods (starting on a Sunday), for which complete sets of measurements had been obtained. We focused on the differences between weekdays and weekends. Data for periods that contained a weekday public holiday or long series of public holidays, such as the New Year’s, Golden Week (29 April to 5 May), or Obon holidays (mid-August), were excluded because we considered that weekly variations might disappear during vacation periods [12]. Moreover, we excluded data associated with sleep periods of less than 3 h or more than 13 h according to the method used in a previous study [8]. As a result, we analyzed the data obtained for 2,914 participants over a total of 24,899 subject-weeks. Table 1 and Table 2 show the participants’ demographic information. Figure 1 shows the number of participants according to gender and age decile.

**Table 1:** Participants’ characteristics (Data are expressed as absolute or mean and SD values).

Number of Participants	2914
Male/Female	2446/468
Age	47.9 ± 11.6

**Table 2:** Participants’ sleep parameters (Data are expressed as absolute or mean and SD values).

Number of Weeks	24,899
Average Nightly Time in Bed (min)	414.2 ± 63.4
Average Nightly Sleep Efficiency (%)	87.3 ± 8.2

### 2.2. Contactless Biomotion Sensor

A contactless biomotion sensor that detects radiofrequency waves was used to determine the subjects’ sleep/wake state based on their bodily and respiratory movements [13,14,15]. It emits radiofrequency wave of 10.525 GHz and the sampling rate of sensor signal is 64 Hz. The sensor outputs two quadrature signals, which represent the I and Q signals. With these two signals, sensor system detects the body movements and respiratory movements separately. Sleep/wake state classified using body movement information after calculating the activity each 30-s epoch, which assessed magnitude and duration of each movement. Respiratory information was used to classify whether person is present or absent. Finally, using above information, the algorithm used by the sensor was developed to classify each 30-s epoch into four categories (sleep/wake/absent/unknown). The “unknown” and “absent” categories signify that nobody was within the range of the sensor, which covers a 45° area and has a detection range of 50–200 cm. System outputs “absent” epochs when there is definitely no respiratory or movement signal detected for several consecutive epochs. The overall accuracies of each 30-s epoch were 84.1% and 85.6%, sensitivities to sleep were 91.8% and 95.3%, sensitivities to wake were 37.6% and 38.9%, and Cohen’s kappa coefficients were 0.31 and 0.53 compared with PSG in the two separate previous validation studies, respectively [14,15].

The contactless biomotion sensor used in this study enables the start and end of the measurement period to be determined in two ways. First, to obtain automatic measurements, the subject sets the start time of the measurement period in advance, and measurements start being taken when the sensor detects the subject. The sensor stops obtaining measurements when the subject has been out of range for a fixed amount of time. Second, manual measurements, in which the subject pushes a button when going to and getting out of bed, can also be obtained. In this study, we used both types of data; however, we excluded data obtained on nights in which an “absent” label was attached to any period (excluded 35,614 weeks, 1304 users). We took this approach to avoid two types of errors: (1) errors caused by the participant being in range of the sensor whilst it was in automatic measurement mode before they actually wanted to go to sleep; and (2) errors caused by the participant forgetting to push the stop button when the sensor was in manual measurement mode.

### 2.3. Web-Based Application

The contactless biomotion sensor makes it possible to upload the data it collects to a web-based healthcare application via a USB or near-field communication connection. Informed consent was obtained from all subjects when they first started to use the web-based application. The subjects were informed about the data confidentiality and handling procedures employed in this study prior to their consent being obtained.

### 2.4. Data Analysis

**Figure 2:**
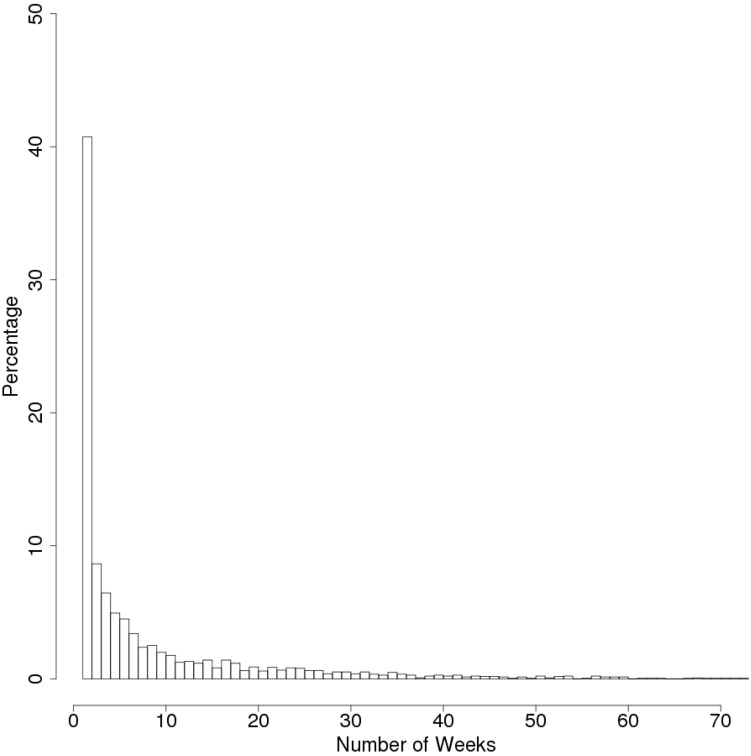
Histogram showing the number of weeks of data available for the subjects according to age decile.

Data were extracted for 7-day periods (starting on Sunday) for which complete sets of measurements had been obtained. The number of weeks of data collected for each participant differed (minimum: 1 week, maximum: 73 weeks). Figure 2 shows a histogram of the distribution of the number of weeks of data collected from the subjects. We examined six sleep parameters in this study: bedtime, wake-up time, time spent in bed (TIB), total sleep time (TST), sleep efficiency (SE), and initial sleep index. TST is the sum of all sleep epochs between sleep onset and waking-up. SE is the ratio of the TST to the time spent in bed multiplied by 100. Initial sleep index is employed to measure the difficulty in falling asleep. It is defined as the lapsed minutes from the bedtime to the first 10-min period where all labels are “sleep” and activity equal to 0. We defined this index since sleep onset latency with activity based algorithm underestimate compared with PSG [14]. In addition, the difference between the maximum MS and median MS (MSmax − MSmed) values detected in each week was used to assess the intra-weekday variation in sleep patterns. In each subject, we randomly selected weeks (maximum 10 weeks, if a subject has only 3 weeks of valid data, selecting all 3 weeks) and then averaging “MSmax − Msmed”. After averaging each subject, mean values were calculated in all subjects. This approach was employed because of the differences in the amount of data available for each participant. One-way ANOVA was used to test for differences in sleep parameters between days or age deciles, and Welch’s *t*-test was used to test for differences between the weekdays and weekends, or genders in each age decile. All statistical analyses were conducted using the software R, version 3.0. 

## 3. Results

### 3.1. All Subjects

Table 3 shows the data collected for each sleep parameter on weekdays (Sunday to Thursday) and weekends (Friday and Saturday). On Friday and Saturday nights, the subjects’ mean bedtime, wake-up time, and MS were delayed by 26, 53, and 40 min, respectively, compared with those seen on Sunday to Thursday nights. As a result, their TIB and TST values were increased by 27 and 22 min, respectively. Welch’s *t*-test detected significant differences in the bedtime, wake-up time, TIB, and TST between weekdays and weekends. Table 4 shows the data collected for each sleep parameter each day. On Sunday nights, the initial sleep index was increased compared with those seen on Tuesday to Friday (Sunday: 42.8 min, Tuesday to Friday: 36.0–37.5 min). ANOVA detected significant differences in the initial sleep index between the day groups (*p* < 0.01).

**Table 3:** Comparison of sleep parameters between weekdays and weekends (Data are expressed as mean ± SD values. Weekdays, from Sunday to Thursday nights; Weekend, Friday, and Saturday nights, Significant difference between weekdays and weekends assessed by Welch’s *t*-test).

Index	Weekdays	Weekend	*p*
Bedtime (HH:MM)	11:46 p.m. ± 87.2	00:12 a.m. ± 102.0	<0.001
Wake-up Time (HH:MM)	06:32 a.m. ± 80.6	07:26 a.m. ± 105.0	<0.001
Sleep Midpoint (HH:MM)	03:09 a.m. ± 77.2	03:49 a.m. ± 96.4	<0.001
Time in Bed (min)	406 ± 65.9	434 ± 75.4	<0.001
Total Sleep Time (min)	354 ± 60.1	376 ± 70.2	<0.001
Sleep Efficiency (%)	87.4 ± 8.2	87.1 ± 8.8	0.089
Initial Sleep Index (min)	38.8 ± 29.5	38.4 ± 33.7	0.613

### 3.2. Differences between the Genders and Age Deciles

**Figure 3:**
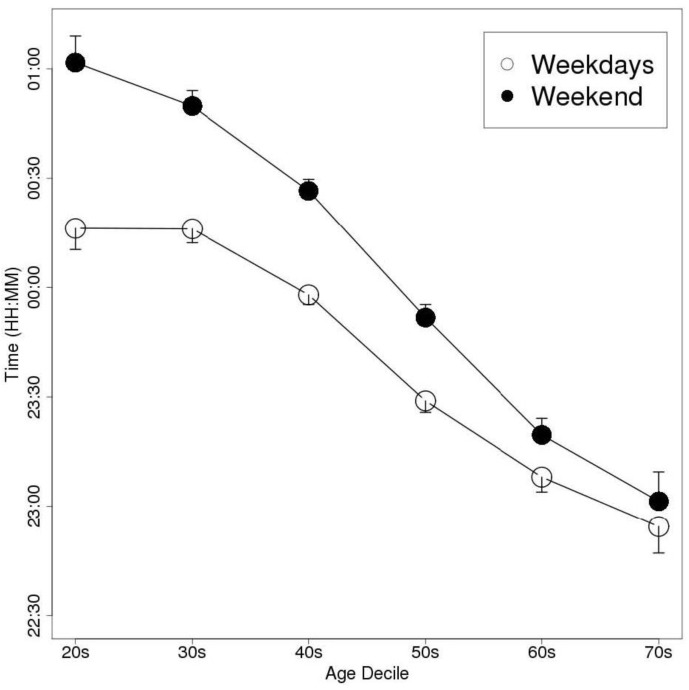
A plot of the subjects’ weekday and weekend bedtimes according to age decile (Data are shown as mean and SEM values).

**Figure 4:**
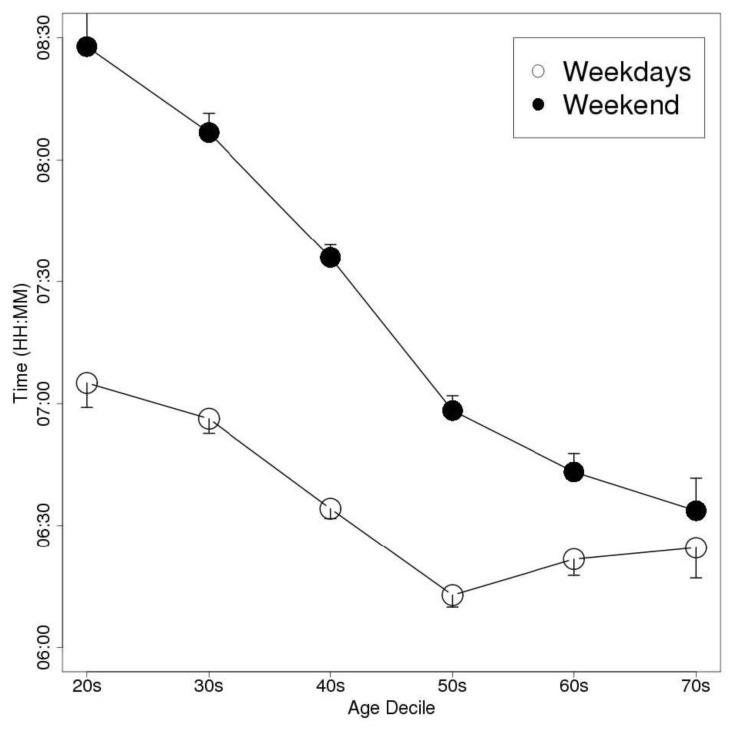
A plot of the subjects’ weekday and weekend wake-up times according to age decile (Data are shown as mean and SEM values).

**Figure 5:**
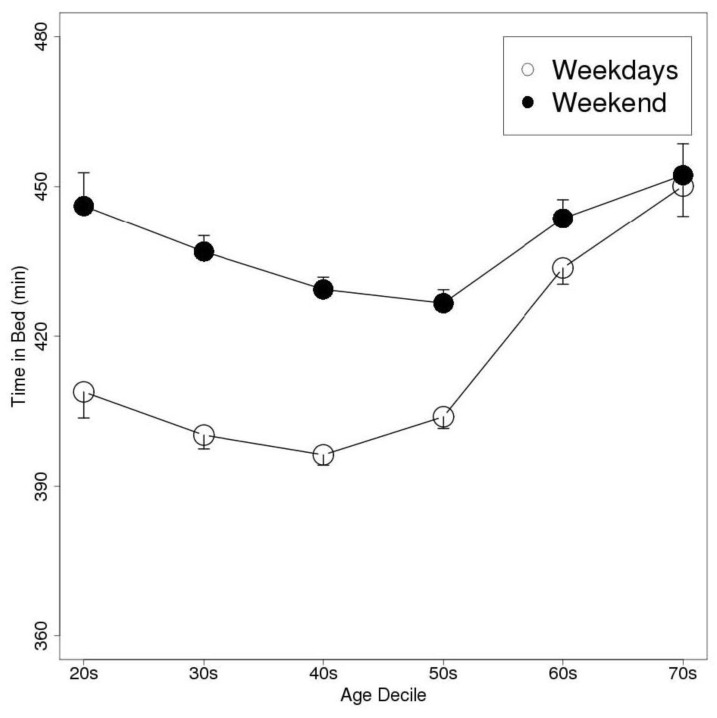
A plot of the subjects’ weekday and weekend TIB values according to age decile (Data are shown as mean and SEM values. TIB, time spent in bed).

**Figure 6:**
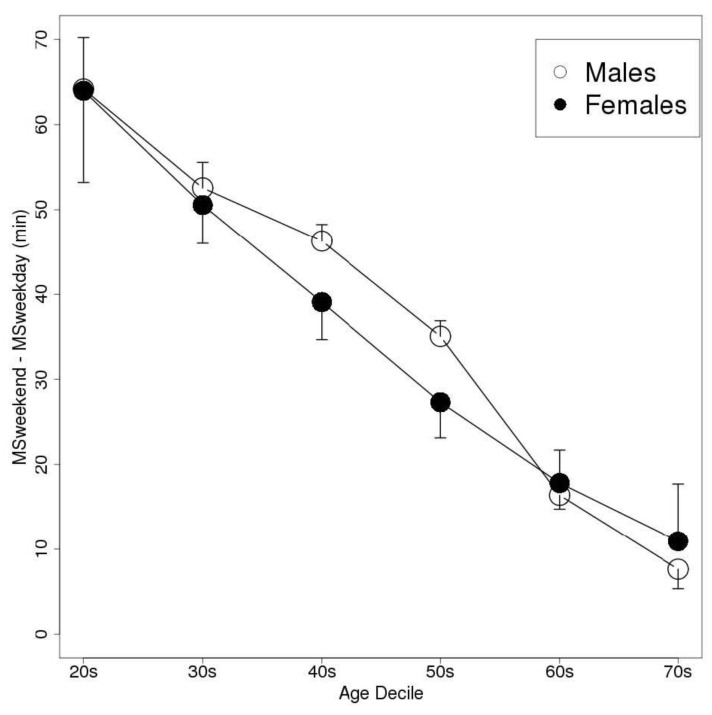
A plot of the subjects’ MSweekend − MSweekday values according to gender and age decile (Data are shown as mean and SEM values. mid-sleep, MS = bedtime + (wake-up time − bedtime)/2).

The weekend delay in the MS was greatest in the individuals in their 20 s, in both men and women. The men delayed their bedtime, wake-up time, and MS by 44, 84, and 64 min, respectively, on the weekend compared with the values recorded on weekdays. As a result, their TIB and TST values were prolonged by 39 and 36 min, respectively. Similarly, the women delayed their bedtime, wake-up time, and MS by 46, 82, and 64 min, respectively, on the weekend, and so their TIB and TST values were increased by 37 and 32 min, respectively. ANOVA detected significant differences in MSweekend − MSweekday between the age groups (*p* < 0.01); however, Welch’s *t*-test did not detect any significant differences in this parameter between the genders. Figure 3, Figure 4 and Figure 5 show the bedtimes, wake-up times, and TIB values, respectively, of each age decile on weekdays and weekends. Figure 6 shows the changes in MSweekend − MSweekday among the genders and age deciles.

**Table 4:** Comparison of sleep parameters among day (Data are expressed as mean ± SD values).

Index	Sunday	Monday	Tuesday	Wednesday	Thursday	Friday	Saturday
Bedtime (HH:MM)	11:40 p.m. ± 96.4	11:44 p.m. ± 93.2	11:47 p.m. ± 93.9	11:48 p.m. ± 92.3	11:51 p.m. ± 96.5	00:15 a.m. ± 107.1	00:09 a.m. ± 109.3
Wake-up Time (HH:MM)	06:33 a.m. ± 87.9	06:32 a.m. ± 86.3	06:31 a.m. ± 86.0	06:33 a.m. ± 84.8	06:34 a.m. ± 87.6	07:21 a.m. ± 109.7	07:31 a.m. ± 112.6
Sleep Midpoint (HH:MM)	03:07 a.m. ± 84.0	03:08 a.m. ± 81.9	03:09 a.m. ± 82.1	03:10 a.m. ± 80.5	03:12 a.m. ± 84.1	03:48 a.m. ±100.0	03:50 a.m. ± 102.5
Time in Bed (min)	413 ± 76.3	408 ± 73.5	404 ± 74.1	405 ±74.0	402 ± 75.3	425 ± 84.1	442 ± 85.1
Total Sleep Time (min)	360 ± 70.2	354 ± 68.3	352 ± 68.6	353 ± 68.6	351 ± 69.1	369 ± 77.7	383 ± 80.5
Sleep Efficiency (%)	87.4 ± 9.1	87.2 ± 9.2	87.5 ± 8.9	87.5 ± 9.1	87.7 ± 8.8	87.1 ± 9.1	87.0 ± 9.5
Initial Sleep Index (min)	42.8 ± 42.2	39.5 ± 37.8	37.5 ± 35.8	37.4 ± 36.3	36.7 ± 34.4	36.0 ± 35.5	40.7 ± 41.7

### 3.3. Differences among Weekdays

On weekdays (from Sunday to Thursday), the difference between MSmax and MSmed was greatest among the subjects in their 20 s (47 min) and smallest among those in their 70 s (35 min). ANOVA detected a significant relationship between MSmax − MSmed and age (*p* < 0.05); however, Welch’s *t*-test did not detect significant differences between the MSmax − MSmed values of men and women among the subjects except in their 60 s (*p* < 0.01). Of the subjects in their 20 s, 22.9% exhibited MSmax – MSmed values of more than 1 h, 12.2% demonstrated bedtime delays of more than 2 h, and 10.3% displayed wake-up time delays of more than 2 h, respectively. These percentages tended to decrease as the age decile increased. Table 5 shows the differences between the subjects’ maximum and median bedtimes, wake-up times, and MS values according to age decile. Table 6 shows the percentage difference between the subject’ maximum and median values over the threshold according to age decile.

**Table 5:** Difference between the maximum and median values of various sleep parameters according to age decile (Data are expressed as mean ± SD values. N, number of participants; MS, mid-sleep; max, maximum value of the parameter on weekdays; med, median value of the parameter on weekdays; BIT, bed in time; BOT, bed out time).

Age Decile	N	MSmax − MSmed	BTmax − BTmed	WTmax − WTmin
20s	165	47 ± 41	61 ± 48	46 ± 57
30s	513	43 ± 42	57 ± 49	44 ± 51
40s	960	44 ± 53	58 ± 61	42 ± 58
50s	793	38 ± 34	52 ± 44	37 ± 45
60s	359	40 ± 34	52 ± 45	42 ± 43
70s	124	35 ± 25	47 ± 40	40 ± 29

**Table 6:** Percentage difference between the maximum and median values of various sleep parameters according to age decile (Data are expressed as mean ± SD values. N, number of participants; MS, mid-sleep; max, maximum value of the parameter on weekdays; med, median value of the parameter on weekdays; BIT, bed in time; BOT, bed out time).

Age Decile	N	MSmax – MSmed ≧ 60 (%)	BTmax – BTmed ≧ 120 (%)	WTmax - WTmed ≧ 120 (%)
20s	166	22.9 ± 1.9	12.2 ± 1.1	10.3 ± 1.0
30s	523	20.3 ± 1.2	10.0 ± 0.7	10.2 ± 1.0
40s	959	19.5 ± 0.8	9.4 ± 0.6	8.1 ± 0.4
50s	810	18.8 ± 0.9	10.2 ± 0.7	7.1 ± 0.3
60s	360	18.0 ± 1.2	8.7 ± 0.8	6.2 ± 0.7
70s	125	18.0 ± 2.2	8.7 ± 1.3	6.7 ± 1.3

## 4. Discussion

This study found that the subjects tended to go to bed and wake-up later on the weekend than on weekdays. As a result, their TIB and TST values were increased because they delayed their wake-up times to a greater extent than their bedtimes. Furthermore, initial sleep index increased on Sunday. The difference between the subjects’ weekday and weekend TIB values was greatest among the individuals in their 20 s and decreased with age. We assume that this trend was indicative of lifestyle changes; *i.e.*, people generally retire around the age of 60. Therefore, they are released from social obligations and accumulate less sleep debt on weekdays so they do not need to alter their sleep patterns on the weekend. Our results show that people in their 60 s and 70 s exhibit higher weekday TIB values than those in their 20 s–50 s.

Previous studies have reported that weekend sleep delays cause disturbances in circadian rhythms. Taylor *et al.* found that waking-up later on both weekend mornings resulted in a delayed circadian rhythm (dim light melatonin onset, DLMO), increased sleep onset latency on Sunday nights, and greater daytime fatigue and sleepiness during the following week [11]. Interestingly, Crowley and Carskadon reported that neither waking-up earlier nor exposure to light on weekend mornings was effective at stabilizing circadian rhythms in younger subjects [16], which indicates the difficulty of recovering from circadian rhythm delays in the short term. Such circadian disturbances also affect reward-related brain functions in healthy adolescents [17]. Furthermore, they are associated with worse academic performance among adolescents with behaviorally-induced insufficient sleep syndrome [18].

In this study, we used MSweekend − MSweekday (which is similar to ΔMS) as an index of sleep pattern delays. Roennberg *et al.* used the MCTQ to examine the sleep patterns of more than 65,000 subjects (primarily central Europeans) and reported that the of the subjects in their 20 s and 50 s exhibited ΔMS values of about 2 h and 1 h, respectively [8]. Another study examined the sleep patterns of subjects with a mean age of 36 using a questionnaire and found that their ΔMS value was about 1 h [19]. Comparing these results with ours suggests that objective ΔMS values might tend to be smaller than subjective ΔMS values. Although it is unclear whether the discrepancy between the abovementioned findings and our results was caused by differences in the subjects’ characteristics or the nature of objective and subjective parameters, trends towards a reduction in ΔMS with age were detected in both our study and the study mentioned above. Previous studies have found that diary-based bedtime and wake-up times were strongly correlated with objective observational data [20,21]; however, there is no evidence to suggest that a correlation exists between data regarding “usual” sleep patterns obtained using a questionnaire and objective daily sleep measurements. Further studies are required to assess the relationships between such values.

Our study also detected intra-weekday variations in sleep patterns during comparisons of the subjects’ maximum and median values for various sleep parameters. Sleep extension and sleep schedule stabilization on the weekend are not sufficient to improve alertness and performance [22]. Our results showed that the greatest mean MSmax − MSmed value was seen in the subjects in their 20s (47 min), and the mean MSweekend − MSweekday value for the same subjects was 64 min. Furthermore, repeated random sampling demonstrated that 22.9% of the subjects in their 20s exhibited mean weekly MSmax − MSmed values of more than 60 min. This indicates that on weekdays one in five of these individuals delayed their bedtime and wake-up time by more than 1 h. This shows that these subjects’ sleep schedules varied on both weekdays and weekends. Although the delays seen on the weekend manifested as delayed wake-up times and extended TIB values, they were primarily caused by the subjects delaying their weekday bedtimes, and hence, getting less sleep because of their obligations on weekday mornings. Soehner *et al.* reported that even minor variations in weekday wake-up times are related to poor subjective sleep quality and that weekday bedtime stability was strongly correlated with consistent weekday wake-up times [23].

Highly regular lifestyles including regular sleep patterns are associated with better subjective sleep in individuals ranging in age from college students to senior citizens [24,25,26]. Monk *et al.* reported that among retired senior citizens the subjects with stable bedtimes and wake-up times (variation of ≤15 min) exhibited better subjective sleep quality and slightly higher SE than the subjects with variable bedtimes or wake-up times (variation of >15 min) [27]. Manber *et al.* manipulated the sleep schedules of irregular sleepers by making them going to bed and wake up within pre-assigned 1-hour time windows, and found that it decreased subjective daytime sleepiness, sleep onset latency, and increased SE [28]. Burgess *et al.* compared the effects of 7–19 nights with a late bedtime (1:00 a.m.) and 7–19 nights with an early bedtime (10:00 p.m.) in an experiment involving a fixed lights on time (7:00 a.m.) and reported that the subjects’ circadian rhythms (DLMO) were significantly delayed in the late bedtime conditions compared with the early bedtime conditions [29]. Clearly, further studies are required to assess the effects of abrupt sleep delays on circadian rhythms and daytime fatigue and sleepiness.

Such delays affect not only sleepiness and daytime performance, but can also influence lifestyle diseases or behaviors. A previous study reported that MSFsc was correlated with body mass index (BMI) among overweight and obese subjects (BMI ≥ 25), but not among individuals with normal body weights (BMI < 25) [8] Circadian misalignment might increase the risk of diabetes and inflammation independent of insufficient sleep [30]. In addition, reduced weekday sleep and weekend oversleeping were found to be associated with suicidality in adolescents [31,32]. Other studies, however, have reported that catching-up on sleep on the weekend decreases the risk of overweightness and hypertension [33,34]. The advantages of getting extra sleep and the disadvantages of the circadian rhythm disturbances caused by weekend catch-up sleep need to be researched further.

Our study has several limitations. First, the subjects might not have been representative of the general public because they bought the contactless biomotion sensor and uploaded the data to the web-based healthcare application of their own volition and so might have had sleep problems or been interested in sleep. Therefore, we can assume that the subjects were motivated to achieve better quality sleep. Second, we analysis data from self-monitoring sleep at home with contactless biomotion sensor, it tend to underestimate wake condition, and then, accuracy of classification decrease with lower SE compared with PSG. Furthermore, although we excluded the data including “absent” state to avoid measurement errors, database may including other types of measurement errors. For example, person wake intentionally after start measurement (e.g., reading book, use smartphone), do something near sensor that it remain forgotten push the stop button (e.g., change clothes, brushing of teeth), go back to sleep after stop measurement. First and second type of errors may overestimate the TIB and wake state (sometime overestimate sleep state, it depends on person’s behavior), detect early bedtime or later wake-up time, third type of error may underestimate the TIB and sleep state, detect early wake-up time. Thus, these measurement errors affect the all of sleep parameters, and may tend to happen weekend than weekdays. Third, although we excluded outliers the database did not contain any information about the subjects’ occupations. Therefore, some of the subjects might have worked on weekends and been off on weekdays. This might explain the differences in ΔMS/MSweekend − MSweekday values between our study and previous studies.

## 5. Conclusions

We detected sleep delays on both weekends and weekdays using home-based contactless biomotion sensors. Such sleep delays were especially prevalent in the subjects in their 20 s–50 s, who were more likely to work on weekdays. This discrepancy reduced with age. The trends detected in this study indicate that the greater social obligations of younger individuals lead to sleep delays on both weekdays and the weekend. Such sleep delays might have an adverse impact on people’s overall quality of life.
